# Satisfaction with care quality and anxiety among family members during nursing home visiting restrictions: The chain mediating effect of emotional regulation and perceived stress

**DOI:** 10.3389/fpubh.2023.1117287

**Published:** 2023-04-06

**Authors:** Zhaozhao Hui, Xiaoqin Wang, Xun Wang, Jinping Zhao, Yunjin Pan, Feng Liu, Ruishi Zheng, Mingxu Wang

**Affiliations:** ^1^School of Public Health, Xi’an Jiaotong University Health Science Center, Xi’an, China; ^2^School of Nursing, Xi’an Jiaotong University Health Science Center, Xi’an, China; ^3^Shaanxi Provincial Hospital of Occupational Disease Control and Prevention, Tongchuan, China; ^4^Songhe Nursing Home, Xi’an Tangcheng Hospital, Xi’an, China

**Keywords:** anxiety, perceived stress, emotion regulation, care quality, nursing home visiting restriction, mediation analysis

## Abstract

**Introduction:**

This study aimed to investigate the psychological well-being (perceived stress and anxiety) of Chinese family members during nursing home visiting restrictions and to elucidate the relationships among satisfaction with care quality, emotion regulation, perceived stress, and anxiety.

**Methods:**

An online survey was conducted with a cross-sectional study design. From 18 to 29 January 2022, a total of 571 family members of nursing home residents completed online questionnaires comprising socio-demographic characteristics, satisfaction with care quality, emotion regulation, perceived stress, and anxiety. Mediation analyses were performed to estimate the direct and indirect effects of satisfaction with care quality on anxiety using the PROCESS macro for SPSS.

**Results:**

The results showed that approximately one-quarter of Chinese family members had anxiety symptoms during nursing home visiting restrictions. Satisfaction with care quality affected anxiety *via* three mediating paths: (a) through cognitive reappraisal (effect = 0.028); (b) through cognitive reappraisal and perceived stress sequentially (effect = −0.057); and (c) through perceived stress (effect = −0.212). The chain mediating effect (path b) accounted for 23.7% of the total effect.

**Conclusions:**

These findings corroborated our hypothesis that cognitive reappraisal (a kind of emotion regulation strategy) and perceived stress mediated the relationship between satisfaction with care quality and anxiety during nursing home visiting restrictions. Efforts to address family members’ psychological well-being by focusing on cognitive reappraisal should be considered.

## 1. Introduction

Coronavirus disease 2019 (COVID-19) has resulted in unprecedented stress on health care systems across the globe (1). The World Health Organization has reported that, as of 5 December 2022, there have been more than 641 million confirmed cases of COVID-19 globally, including 6, 621,060 deaths (2). Older adults are more likely to contract this disease and having comorbidities places them at a higher risk of worse outcomes (e.g., complications, intensive care unit admissions, death) compared with general populations (3). In particular, nursing homes are an important, high-risk target for emerging pathogens due to the presence of vulnerable residents and frequent visitors (4). For long-term care facilities, the World Health Organization therefore has announced special infection and prevention control precautions against COVID-19, including visiting restrictions (5, 6).

The visiting restriction measures undoubtedly can prevent the SARS-CoV-2 transmission in nursing homes, meanwhile, it introduces negative psychosocial impacts not only for the residents but also for their family members (7, 8). For example, a qualitative study revealed that nursing home residents and their family members experienced anxiety, severe stress, and grief during the visiting restriction periods (7). Moreover, a cross-sectional survey found that family members of nursing home residents with cognitive impairment reported significantly lower psychosocial and emotional well-being during the COVID-19 lockdown (8). Family members often stay involved in the caregiving role after institutionalization (9) and meaningful family involvement can potentially improve the quality of life of their loved ones who reside in nursing homes (10). The traditional way for family members to be involved is through in-person visits, by which, for example, they can ensure the care quality of their older relatives and maintain family relationships (11). Previous evidence has shown that family members visit more frequently and provide more hands-on assistance when they are concerned about the adequacy of care in nursing homes (12). Family members are reported to be frustrated about not being able to touch their older relatives or participate in their care due to visiting restrictions (7). Nevertheless, the relationship between the satisfaction with care quality and psychological well-being of family members during visiting restrictions remains to be investigated.

Emotion regulation refers to the process by which individuals modify their emotions, their response to the emotions or the situations that elicit emotions in order to respond appropriately to environmental demands (13). In the Stress and Coping Model, individuals use an array of coping strategies to manage specific external and/or internal demands that are appraised as stressful, including regulating emotional responses to the problem (emotion-focused coping) and managing the problem causing the distress (problem-focused coping) (14). The coping processes thereby affect the social, psychological, and/or somatic outcomes of stressful encounters (14). In previous studies, emotion regulation has been proposed as a vital mediator of stress adjustment (15, 16) and links between inappropriate or maladaptive emotion regulation strategies and anxiety had been found in different populations (17–21). Amidst the COVID-19 pandemic, it is of great importance to actively take control of one’s emotions to cope with this invisible enemy (22, 23). Effective regulation of emotions can reduce negative emotions, enhance well-being (22), and predict quality of life decline (23). Exploring the role of emotion regulation in promoting psychological well-being can provide significant evidence for developing potential interventions and support services, however, its effect in the relationships of satisfaction with care quality, perceived stress, and anxiety has not yet well understood among Chinese family members of nursing home residents during the visiting restrictions.

This cross-sectional study, therefore, aimed to investigate the level of anxiety in Chinese family members of nursing home residents during visiting restrictions due to COVID-19 pandemic and to elucidate the relationships among satisfaction with care quality, emotion regulation, perceived stress, and anxiety. The hypotheses of this study were as follows: (a) Chinese family members of nursing home residents experienced anxiety symptoms during visiting restrictions; (b) Those who were less satisfied with the care quality had a greater level of anxiety; and (c) Emotion regulation and perceived stress played a chain mediating effect in the relationship between satisfaction with care quality and anxiety.

## 2. Methods

### 2.1. Procedure

A cross-sectional study was conducted in family members of nursing home residents from 18 to 29 January 2022. Nursing homes that met the following criteria were recruited: (a) located in Shaanxi province, China, (b) provided both custodial care and skilled care to the residents, and (c) was implementing the policy of visiting restrictions due to COVID-19. With a convivence sampling method, we approached a total of 10 private nursing homes and all their superintendents agreed to participate in this study. The nursing home staff briefly introduced the objectives and procedures of this study to the potentially eligible participants and asked whether they had an interest in participation. Those who agreed to voluntarily participate in this study would complete an online questionnaire anonymously *via* the Sojump platform. To be eligible, the family members had to: (a) be aged 18 years or older, (b) have an older relative (aged 60 years or older) living in the nursing homes, and (c) invest the most hours into the care of their older relative after institutionalization. Those who refused to participate were excluded from this study.

### 2.2. Measurements

#### 2.2.1. Socio-demographic characteristics

The socio-demographic characteristics (e.g., age, gender, educational level, marital status) of both the family members and their older relatives were collected *via* a self-administrated questionnaire. For family members, their average visiting frequency before nursing home visiting restrictions was surveyed. For residents, the length of stay, number of children, chronic diseases, and disability of activities of daily living (ADL) were collected. Additionally, the relationship between the family member and his/her older relative was surveyed.

#### 2.2.2. Satisfaction with care quality

Satisfaction with care quality was assessed by a single self-reported question: how much are you satisfied with the care quality in the nursing home during the visiting restriction period? Family members responded to this question with answers on a 5-point Likert scale, ranging from 1 (very dissatisfied) to 5 (very satisfied).

#### 2.2.3. Emotion regulation

Emotion regulation was assessed by the 10-item Emotion Regulation Questionnaire (ERQ) (24). The ERQ was designed to measure two emotion regulation strategies, namely cognitive reappraisal (6 items) and expressive suppression (4 items). Family members answered each item from 1 (strongly disagree) to 7 (strongly agree). Each facet’s scoring is kept separate. The total score ranged from 6 to 42 for cognitive reappraisal and from 4 to 28 for expressive suppression, with a higher score indicating more tendency to use the corresponding emotion regulation strategy. In the current study, the Cronbach’s α coefficient for cognitive reappraisal and expressive suppression was 0.951 and 0.906, respectively.

#### 2.2.4. Perceived stress

Perceived stress was assessed by the 4-item Perceived Stress Scale (PSS-4) (25). Family members answered each item from 0 (never) to 4 (very often). The total score of PSS-4 was obtained by reversing the scores on the positive items (items 2 and 3) and then summing across all the items (ranges: 0–16). The higher the score, the more perceived stress. The PSS-4 score of 6 or more indicates a high level of perceived stress (26). In the current study, the Cronbach’s α coefficients for the negative items and positive items were 0.828 and 0.870, respectively.

#### 2.2.5. Anxiety

Anxiety was assessed by the 7-item Generalized Anxiety Disorder (GAD-7) (27). Each item was rated on a 4-point Likert scale from 0 (not at all) to 3 (nearly every day). The total score of GAD-7 was computed by summing the ratings on all items (ranges: 0–21). A higher score indicates more anxiety. The severity of anxiety can be divided into minimal (0–4), mild (5–9), moderate (10–14), and severe (15–21). In the current study, the Cronbach’s α coefficient for GAD-7 was 0.957.

### 2.3. Statistical analysis

Statistical analyses were performed by using the software SPSS 25.0 for Windows (IBM Corp., Armonk, NY, United States). Descriptive analyses were conducted to summarize the study variables, and Cronbach’s α coefficients were calculated for the questionnaires used (ERQ, PSS-4, GAD-7). Correlation analyses were conducted to examine the bivariate correlations between the main variables (i.e., satisfaction with care quality, emotion regulation, perceived stress, and anxiety). The mediation analysis was performed by using PROCESS macro (Model 6) for SPSS (28). Variables related to the independent (satisfaction with care quality) and dependent (anxiety) variables were adjusted as confounders. Ordinary least-squares framework was used to estimate the total effect, direct effect, and indirect effect, with 5,000 bias-corrected bootstrap resamples. The significance of the effects was evaluated with Sobel test. The 95% confidence intervals (CI) were calculated to determine whether mediating variables helped explain the relationship between independent and dependent variable. If the 95% CI did not include zero, it indicated that the effect was statistically significant. The proportion mediated was calculated by dividing the indirect effect by the total effect. The significance level in the current study was set at 0.05 (two-tailed).

## 3. Results

### 3.1. Basic characteristics of the participants

A total of 626 family members of nursing home residents were approached, but 615 were deemed as eligible. Questionnaires were sent to the 615 family members, of which nine refused to participate in and 35 completed the survey faster than 2 s per item (effective response rate = 92.8%). A sample of 571 family members and their older relatives, therefore, was finally included and analyzed in this study. The basic characteristics of the included participants are demonstrated in Table 1. The family members aged from 21 to 83 years (Mean = 42.4, SD = 12.56). Most of them were female (69.2%), obtained a bachelor’s degree or above (63.6%), and were married (80.9%). For the family-resident relationships, most of the participants were sons (*n* = 146, 25.6%) or daughters (*n* = 223, 39.1%). Regarding the visiting frequency before visiting restrictions, 77.9% of the family members visited their older relatives at least two to three times per month.

**Table 1:** Basic characteristics of the participants in this study (*N* = 571).

Variables		*n*	%
Family members			
Age^a^		42.4 ± 12.56	
Gender	Male	176	30.8
	Female	395	69.2
Education level	Junior high school or below	60	10.5
	Senior high school	148	25.9
	Bachelor or above	363	63.6
Marital status	Married	462	80.9
	Single/Divorced/Widowed	109	19.1
Relationships	Son	146	25.6
	Daughter	223	39.1
	Grandchild	79	13.8
	Others	123	21.5
Visiting frequency	Almost everyday	52	9.1
	Every 2 or 3 days	82	14.4
	Weekly	202	35.4
	Two to three times per month	109	19.1
	Monthly	61	10.7
	Less than monthly	65	11.4
Anxiety (GAD-7 scores)	Minimal (0–4)	430	75.3
	Mild (5–9)	99	17.3
	Moderate (10–14)	23	4.0
	Severe (15–21)	19	3.3
Residents			
Age^a^		77.6 ± 8.48	
Gender	Male	281	49.2
	Female	290	50.8
Length of stay	<3 months	100	17.5
	3–6 months	132	23.1
	6–12 months	121	21.2
	1–3 year(s)	136	23.8
	>3 years	82	14.4
Education level	Primary school and below	193	33.8
	Junior high school	159	27.8
	Senior high school	150	26.3
	Bachelor and above	69	12.1
Marital status	Married	289	50.6
	Single/Divorced/Widowed	282	49.4
Number of Children	Null	28	4.9
	1	83	14.5
	2	215	37.7
	3	162	28.4
	>3	83	14.5
Chronic disease	Yes	448	78.5
	No	123	21.5
ADL disability	No	217	38.0
	Slight	179	31.3
	Moderate	99	17.3
	Severe	68	11.9
	Unclear	8	1.4

ADL, activities of daily living.

a The mean and standard division are presented.

The average age of the residents was 77.6 years (SD = 8.48, range: 60–100 years). Half of the residents were female. Only 12.1% of the residents achieved a bachelor’s degree or above. Most of the residents lived in the nursing homes for no more than 1 year (61.8%) and had two or three children (66.1%). Those who were married accounted for half of the total sample (50.6%), while single, divorced, or widowed for another half (49.4%). Totally 78.5% of the residents were reported to have chronic diseases and 60.5% had ADL disability.

### 3.2. Bivariate correlations

As seen in Table 2, the perceived stress of family members scored at 6.27 on average (SD = 2.68), while the median score for anxiety was 1.00 (interquartile range = 4.00). According to the GAD-7 scoring criteria, 17.3, 4.0, and 3.3% of the family members experienced mild, moderate, and severe anxiety, respectively. Correlation analyses showed that satisfaction with care quality was positively correlated with cognitive reappraisal (*r* = 0.122, *p* < 0.01) but negatively related to the perceived stress (*r* = −0.162, *p* < 0.01) and anxiety scores (*r* = −0.162, *p* < 0.01). Cognitive reappraisal was negatively correlated with perceived stress (*r* = −0.233, *p* < 0.01), meanwhile, perceived stress was positively associated with anxiety (*r* = 0.204, *p* < 0.01).

**Table 2:** Correlations of satisfaction with care quality, emotion regulation, perceived stress, and anxiety^a^.

	Mean ± SD	Skewness	Kurtosis	1	2	3	4	5
1. Satisfaction with care quality	5.00 (1.00)^b^	−2.114	4.448	1.000				
2. Cognitive reappraisal (ERQ)	30.38 ± 9.82	−0.848	0.076	0.122**	1.000			
3. Expressive suppression (ERQ)	17.58 ± 6.95	−0.306	−0.686	0.074	0.705**	1.000		
4. Perceived stress (PSS-4)	6.27 ± 2.68	−0.492	0.319	−0.162**	−0.233**	0.027	1.000	
5. Anxiety (GAD-7)	1.00 (4.00)^b^	2.062	4.379	−0.162**	−0.070	0.024	0.204**	1.000

SD, standard deviation; ERQ, Emotion Regulation Questionnaire; PSS, Perceived Stress Scale; GAD, Generalized Anxiety Disorder.

a Pearson correlation analyses were conducted among cognitive reappraisal, expressive suppression, and perceived stress, while Sperman correlation analyses were conducted for satisfaction with care quality and anxiety.

b The median and the interquartile range are presented.

** *p* < 0.01.

### 3.3. Mediation analyses

**Figure 1:**
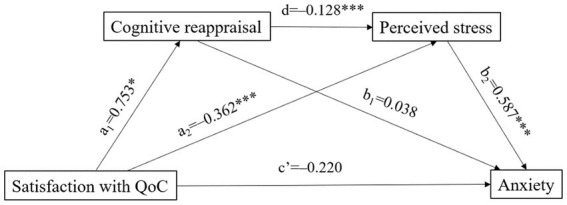
The chain mediation analyses of cognitive reappraisal and perceived stress on anxiety (ADL disability of the residents, age of the family members, and expressive suppression were included as covariates. QoC, quality of care, *p < 0.05, ***p < 0.001).

Results of the mediating effects of cognitive reappraisal and perceived stress between the relationship of satisfaction with care quality and anxiety are illustrated in Figure 1 and Table 3. After controlling for the ADL disability of residents, the age of family members, and expressive suppression, satisfaction with care quality did not directly affect anxiety (*c*’ = −0.220, *p* = 0.217); however, it had a direct and significant positive prediction on cognitive reappraisal (*a*_1_ = 0.753, *p* < 0.05) and could directly and negatively predict the level of perceived stress (*a*_2_ = −0.362, *p* < 0.001). Cognitive reappraisal negatively predicted perceived stress (*d* = −0.128, *p* < 0.001), which further had a positive prediction on anxiety (*b*_2_ = 0.587, *p* < 0.001) (Figure 1).

**Table 3:** Mediating model examination by bootstrap.

	Effect	Boot SE	Boot LLCI	Boot ULCI
Total effect	−0.240	0.708	−0.394	−0.115
Direct effect	−0.220	0.178	−0.570	0.130
Ind1	0.028	0.020	0.001	0.084
Ind2	−0.057	0.025	−0.111	−0.013
Ind3	−0.212	0.069	−0.369	−0.092

SE, standard error; LLCI, lower limit confidence interval; ULCI, upper limit confidence interval.

Ind1: Satisfaction with care quality→Cognitive reappraisal→Anxiety.

Ind2: Satisfaction with care quality→Cognitive reappraisal→Perceived stress→Anxiety.

Ind3: Satisfaction with care quality→Perceived stress→Anxiety.

The bootstrap examination results showed that satisfaction with care quality affected anxiety *via* three indirect paths, for which the total effect was −0.240. In path 1, satisfaction with care quality affected anxiety through cognitive reappraisal (effect = 0.028, 95% CI: 0.001, 0.084). In path 2, satisfaction with care quality had an influence on anxiety through cognitive reappraisal and perceived stress sequentially (effect = −0.057, 95% CI: −0.111, −0.013), which accounted for 23.7% of the total effect. In path 3, satisfaction with care quality affected anxiety through perceived stress (effect = −0.212, 95% CI: −0.369, −0.092), accounting for 88.3% of the total effect (Table 3). These findings corroborated our hypothesis that cognitive reappraisal and perceived stress mediated the relationship between satisfaction with care quality and anxiety. Since pre-pandemic visiting frequency of family members may influence their satisfaction with care quality, emotion regulation, perceived stress, and anxiety during visiting restriction, we conducted a stratified analysis by visiting frequency (less than once a week, more than once a week) and similar results were obtained.

## 4. Discussion

This study investigated anxiety symptoms among Chinese family members of nursing home residents during visiting restrictions and examined its relationships with satisfaction with care quality. We found that approximately one-quarter of the family members of nursing home residents had anxiety symptoms during visiting restrictions. More importantly, our results demonstrated that cognitive reappraisal (a kind of emotion regulation strategy) and perceived stress played a chain mediating role in the relationship between satisfaction with care quality and anxiety, which provides a new perspective for the purpose of promoting the psychological well-being of family members during nursing home visiting restrictions.

We found that approximately one-quarter of the family members had anxiety symptoms, which is comparable to the prevalence of anxiety in general populations during the COVID-19 pandemic reported in a recent meta-analysis of 43 studies (29). Moreover, the mean score for PSS-4 was 6.27, suggesting a high level of self-perceived stress in family members of nursing home residents during visiting restrictions (26). Our findings are in line with a previous qualitative study, in which both residents and their family members experienced anxiety and severe stress due to isolation and distancing during the COVID-19 pandemic (7). The reason why family members felt stressed and anxious can be the inability to visit their old relatives in persons due to the social distancing policies (30). In addition, this study revealed that satisfaction with care quality was negatively related to both perceived stress and anxiety among family members of nursing home residents. Family members generally expected nursing homes to provide high-quality care and support the well-being of their loved ones (31). A previous study found that the quality of nursing home personal care was a major source of stress for family members (32), which echoes the results of the current study.

For the mechanisms of how satisfaction with care quality affects anxiety in family members of nursing home residents, we found three indirect paths although there were no direct effects. Firstly, cognitive reappraisal positively mediated the relationship between satisfaction with care quality and anxiety. Individuals who tend to use cognitive reappraisal are more likely to interpret stressful events in a more optimistic way and make active efforts to repair negative moods (33). A randomized controlled trial reported that cognitive reappraisal as a brief online intervention could ease acute stress and strengthen the mental health of parents during the COVID-19 pandemic (34). In addition, family members who experienced a higher level of satisfaction with care quality tended to utilize the cognitive reappraisal strategy, which further activates the brain structure (e.g., amygdala) and leads to less anxiety (35). Our study found that family members of nursing home residents scored cognitive reappraisal at a relatively lower level when compared with previous studies (36, 37). This suggests that cognitive reappraisal skills should be cultivated for family members during nursing home visiting restrictions.

Secondly, satisfaction with care quality indirectly affects anxiety through perceived stress, which is the primary path that accounted for 88.3% of the total effect. This mediating role of perceived stress to anxiety is partially consistent with previous studies in other populations (38–40). In college students, perceived stress mediates the association between sleep quality and anxiety symptoms (38) as well as the relationship between facing existential issues (loneliness and death) and anxiety symptoms (39). During the COVID-19 pandemic, Pradhan et al. examined the fear of death among young adults and found that neuroticism positively correlated to death anxiety but this relationship was completely mediated by perceived stress (40). In a qualitative study, almost all family members of people with dementia expressed stress when they were worried about the care quality in nursing homes (41). Findings of the current study verified that family members with higher satisfaction with care quality would experience a lower level of perceived stress and tend to undergo fewer anxiety symptoms.

Thirdly, cognitive reappraisal and perceived stress exerted a chain mediating effect between satisfaction with care quality and anxiety. This path illustrated that cognitive reappraisal acted as a partial mediator between satisfaction with care quality and perceived stress while perceived stress fully mediated the relationship between cognitive reappraisal and anxiety. Family members who were more satisfied with care quality would adopt the cognitive reappraisal strategy more frequently (*β* = 0.753), which negatively predicted perceived stress (*β* = −0.128). Meanwhile, the lower level of perceived stress, the fewer anxiety symptoms (*β* = 0.587). These results are consistent with the basic viewpoint of the Stress and Coping Model (14). That is, when faced with a stressful encounter, the individual would mobilize coping efforts, which can influence the perception of stress and thereby lead to psychological outcomes (e.g., anxiety) (14). Cognitive reappraisal is generally viewed as a healthy emotion regulation strategy since it attempts to reinterpret an emotion-eliciting situation in a way that alters its meaning and changes its emotional impact (24). This path provided a deeper understanding of the mechanisms of how satisfaction with care quality affects anxiety and reemphasized the vital role of cognitive reappraisal in alleviating anxiety symptoms of family members during nursing home visiting restrictions. Cognitive reappraisal techniques, such as positive reframing, self-distancing, and temporal distancing (42), can be implemented for family members during visiting restrictions. By this way, both the perceived stress and anxiety of family members could be alleviated. In addition, high-quality care and family-resident communication cannot be ignored to minimize the negative effects of nursing home visiting restrictions. It is suggested that feasible and acceptable digital solutions, such as web conferencing, can be promoted in nursing homes during this special period (43). Policies limiting of visitation isolated the family members from their relatives who lived in nursing homes, such interventions can allow family members access to residential care and may further improve their satisfaction with care quality.

To our knowledge, this is the first study to quantitatively investigate the psychological well-being among Chinese family members of nursing home residents during visiting restrictions. The findings contribute to a deeper understanding of the mechanism of how satisfaction with care quality affects anxiety. However, this study had several limitations that should be mentioned for future research. Firstly, this study was a cross-sectional design in which the interpretation of the direction of associations has been clouded and cause-effect relationships among the variables cannot be deduced. It is suggested to employ interventional experiments or longitudinal studies to validate the mediating effects and provide a better understanding of satisfaction with care quality and anxiety in the future. In addition, the satisfaction of care quality was measured by a single self-reported question, which may not reflect the actual care quality in nursing homes. However, subjective perception is always of great importance in psychological studies. Although the residents were provided with high-quality care in nursing homes, their family members can still experience a low level of psychological well-being (e.g., anxiety) if they perceived the care as dissatisfying. Moreover, the results of this study were not compared to the nursing home situation before visitation restrictions. Some older adults had been living in the nursing homes for a long time, family members may recall the care quality prior to the COVID-19 pandemic when evaluating their satisfaction with care quality. Finally, this study was carried out with a Chinese sample during the COVID-19 lockdown, the applicability of the study results to the populations in other countries should be further validated.

## 5. Conclusion

The results of this study demonstrate that approximately one-quarter of the family members of nursing home residents experienced anxiety symptoms during visiting restrictions. In addition, the relationship of satisfaction with care quality and anxiety is mediated by cognitive reappraisal (a kind of emotion regulation strategies) and perceived stress, which provides significant evidence for developing potential interventions and support services. Efforts to address family members’ psychological well-being by focusing on cognitive reappraisal should be considered.

## Data availability statement

The raw data supporting the conclusions of this article will be made available by the authors, without undue reservation.

## Ethics statement

The studies involving human participants were reviewed and approved by the Biomedical Ethics Committee of Xi’an Jiaotong University Health Science Center (2022-0004). The patients/participants provided their written informed consent to participate in this study.

## Author contributions

ZH, XQW, and MW contributed to the conception and design of this study. FL and RZ contributed to data acquisition. ZH and XW performed the statistical analysis. ZH, XW, JZ, and YP interpreted the data and drafted the manuscript. All authors contributed to the critical revision of the manuscript and approved the version for publication.

## Funding

This study was supported by the Fundamental Research Funds for the Central Universities (SK2023004).

## Conflict of interest

The authors declare that the research was conducted in the absence of any commercial or financial relationships that could be construed as a potential conflict of interest.

## Publisher’s note

All claims expressed in this article are solely those of the authors and do not necessarily represent those of their affiliated organizations, or those of the publisher, the editors and the reviewers. Any product that may be evaluated in this article, or claim that may be made by its manufacturer, is not guaranteed or endorsed by the publisher.

## Abbreviations

COVID-19: coronavirus disease 2019
ADL: activities of daily living
ERQ: Emotion Regulation Questionnaire
PSS: Perceived Stress Scale
GAD: Generalized Anxiety Disorder
